# Hot Spots and Trends in the Relationship between Cancer and Obesity: A Systematic Review and Knowledge Graph Analysis

**DOI:** 10.3390/life13020337

**Published:** 2023-01-27

**Authors:** Le Gao, Tian Yang, Ziru Xue, Chak Kwan Dickson Chan

**Affiliations:** 1 Faculty of Intelligent Manufacturing, Wuyi University, Jiangmen 529000, China; 2 Institute for Guangdong Qiaoxiang Studies, Wuyi University, Jiangmen 529000, China; 3 Faculty of Social Sciences, Lingnan University, Hongkong 999077, China

**Keywords:** cancer, tumor, obesity, knowledge graph, visualization

## Abstract

Cancer is one of the most difficult medical problems in today’s world. There are many factors that induce cancer in humans, and obesity has become an important factor in inducing cancer. This study systematically and quantitatively describes the development trend, current situation and research hotspot of the relationship between cancer and obesity by using document statistics and knowledge graph visualization technology. Through the visualization technology analysis of knowledge graph in this study, the research hotspot and knowledge base source of the relationship between cancer and obesity in the last 20 years have been ascertained. Obesity-related factors, such as immunity, insulin, adiponectin, adipocytokines, nonalcoholic fatty liver and inflammatory reaction, may affect the occurrence of obesity and increase the risk of cancer. Obesity-related cancers include respiratory cancer, colorectal cancer, hepatocellular cancer, prostate cancer, gastric cancer, etc. Our research provides direction and basis for future research in this field, as well as technical and knowledge basis support for experts and researchers in related medical fields.

## 1. Introduction

**Figure 1:**
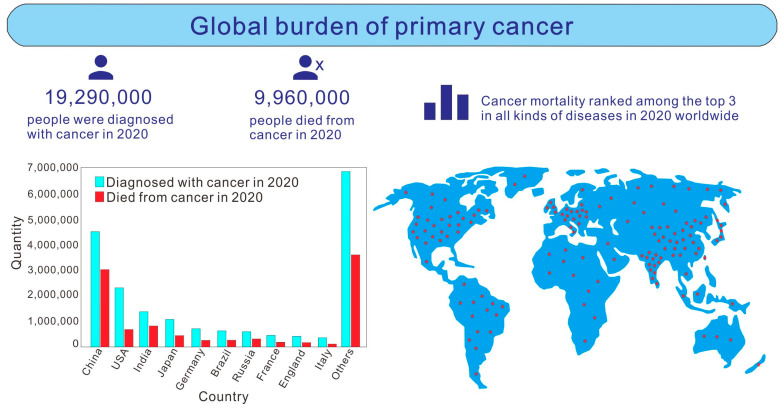
Development trend of cancer patients in the world.

Cancer is one of the most difficult medical problems in the world today. It is a public health problem that seriously threatens human life and health [1,2,3]. According to research statistics [4,5,6], among various diseases, the mortality rate of malignant tumor is the second highest, second only to cardiovascular and cerebrovascular diseases. In the last 20 years, the incidence rate and mortality rate of cancer have been rising at a rate of 20% each year. Figure 1 shows the development trend of cancer patients in the world. The World Health Organization’s International Agency for Research on Cancer (IARC) has released data on the global burden of cancer for 2020, which looks at incidence and mortality rates for 36 cancer types in 185 countries. As can be seen from the picture, in 2020, there will be 19,290,000 new cancer patients and 9,960,000 deaths worldwide. The top 10 countries in the number of cancer patients are China (4,568,754, 23.7%), the United States (2,281,658, 11.8%), India (1,324,413, 6.9%), Japan (1,028,658, 5.3%), Germany (628,519, 3.3%), Brazil (592,212, 3.1%), Russia (591,371, 3.1%), France (467,965, 2.4%), England (457,960, 2.4%) and Italy (415,269, 2.2%). Others (6,936,010, 36%) refer to the number of cancer patients in 175 other countries, including Canada, Indonesia, Mexico, Iran and Turkey, which also have a large number of cancer patients. Due to the increasing variation in the number of people in each country, countries with a larger population base in this study also had more cancer cases. According to the statistics in the literature [7], due to the different genital systems and endocrine levels of males and females, there will be significant differences in cancer prevalence between the sexes. For example, women get metrocarcinoma, ovarian cancer and carcinoma of vulva. In men, prostate cancer occurs and carcinoma of the testis occurs. Breast cancer also varies by gender, with 99.07% of occurrence in women and 0.93% in men. In oral cancer and pharyngolaryngeal cancer, the proportions of males and females were 71.67% and 28.33%, respectively. The proportion of male and female thyroid cancer patients was 28.95% and 71.05%, respectively. In cancers of the digestive system, such as esophagus cancer, gastric cancer, intestinal cancer, colon cancer, rectum cancer, liver cancer, gallbladder carcinoma and pancreatic cancer, the proportion was 56.36% male and 43.64% female, respectively. Among respiratory cancers, such as laryngocarcinoma and lung cancer, 51.58% were male and 48.42% were female, respectively. Among the patients with bone cancer, 55.24% were male and 44.76% were female. Among skin cancer patients, 57.91% were male and 42.09% were female. Among leukemia patients, 59.04% were male and 40.96% were female. With the continuous development and progress in science and technology and medical technology, cancer treatment methods, such as surgery, chemotherapy and cell-based therapy, have made great progress [8,9,10]. However, due to the complex pathogenesis of cancer, it is very difficult to treat it. Therefore, cancer prevention and search for efficient and low recurrence cancer treatment methods have always been difficult and topical issues in the field of cancer prevention and treatment.

Cancer is caused by many factors, including genetic factors, endocrine factors, immune factors, lifestyle (smoking), environmental pollution (diet, air), ionizing radiation, etc. A large survey in the internationally famous medical journal *The Lancet* shows that obesity and being overweight will increase the risk of cancer, and body mass index (BMI) (weight divided by the square of height) is significantly related to a variety of cancers [11,12,13]. Obesity is usually accompanied by a series of diseases, such as hypertension, hyperlipidemia, coronary heart disease, diabetes, cardiovascular and cerebrovascular diseases, and endocrine diseases, which have a huge impact on people’s health [14,15,16,17,18,19]. The main reason why obese people are prone to cancer is that patients often have hyperinsulinism and hyperlipidemia. Hyperlipemia reduces the immunity of the body, so that the ability of immune cells to recognize and kill tumor cells will be relatively weak, so the incidence rate of cancer has increased accordingly [20]. An article [12] published in the medical journal *The Lancet* tallied a population-based cohort study of 5.24 million UK adults. The analysis showed that overweight and obese people were significantly more likely than the general population to develop the following 10 types of cancer. According to the ranking, they are: cervical cancer (62%), gallbladder (31%), kidney (25%), liver (19%), colorectal cancer (10%), cervical cancer (10%), thyroid cancer (9%), leukemia (9%), ovarian cancer (9%) and breast cancer (5%). In order to deeply explore the correlation between cancer and obesity and the research hotspots and trends in recent years, this study will use knowledge mapping technology to analyze and discuss the literature.

Knowledge graph is essentially a large-scale semantic network, which is rich in concepts, entities and semantic relationships [21,22]. As a semantic network, knowledge atlas is an important method of knowledge representation in the age of big data. As a technical system, knowledge map is representative of progress in knowledge engineering in the age of big data [23]. In recent years, knowledge mapping technology has been widely used in various fields [24,25,26,27,28,29,30,31,32]. It can show the development and structural relationship of scientific knowledge through graphics and can create the mathematical expression that reveals scientific knowledge and laws to graphical expression. The knowledge map has gradually become an important tool for grasping the development trend of the discipline, the main research direction and technology-aided decision making.

By studying the literature on the causes of cancer, this study found that obesity is an important factor in cancer. In the study of cancer diseases, obesity and its synonyms, such as being overweight and fat, etc., appear very frequently. In addition, *The Lancet*, an influential journal in this research field, also came to the same conclusion that the increase in body mass index is correlated with the increase in tumor incidence, that is, obesity is closely related to the risk rate of cancer [11]. Influential research has clearly demonstrated a strong link between cancer and obesity, which is the value of this study. In the recent decades, researchers and medical experts in medical academia have carried out a large number of experiments and studies on the relationship between obesity and cancer. They have accumulated a large number of objective and valuable results. However, there are few systematic and comprehensive review studies on this basis using computer technology. Based on this, this paper will use knowledge graph analysis method to conduct quantitative visual analysis and discussion on the academic literature of the relationship between cancer and obesity in the past 20 years, so as to clarify the research hotspot and trend of the relationship between cancer and obesity, as well as providing technical support and knowledge foundation for medical experts and researchers in this field in the future.

## 2. Materials and Methods

### 2.1. Data Source and Search Strategy

**Figure 2:**
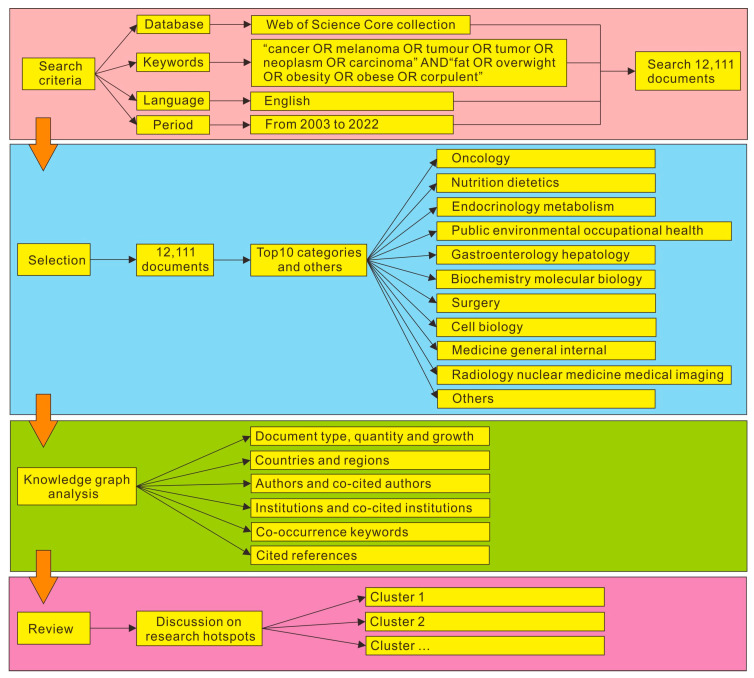
The overall research framework.

**Figure 3:**
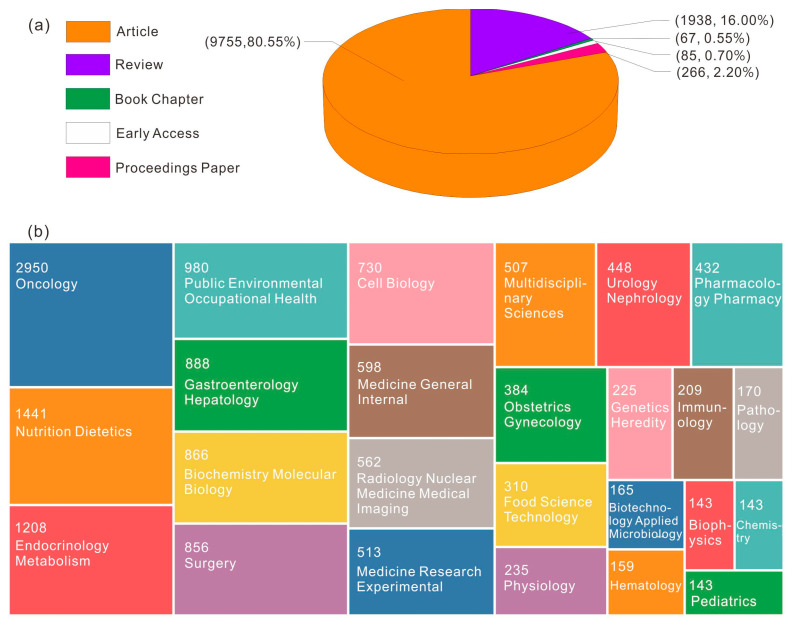
(**a**) Literature types; (**b**) Subject categories.

The data used in this paper were from the Web of Science core collection database (WoS). WoS is one of the most comprehensive bibliographic sources available, which frequently provides users an online access port to scientific literature with high quality, where we can get access to the cited references. Many scholars use WoS database when performing bibliometric analysis. For example, the authors in [33] used WoS database to conduct bibliometrics and visualization analysis of neuropathic pain articles in the past 20 years to explore the disciplinary hot spots and trends of this study. In [34], the authors also used WoS database to conduct a comprehensive bibliometric analysis of scientific research achievements of sickle cell disease and explored the global research situation and historical progress in sickle cell disease. Figure 2 shows the research framework of searching literature knowledge using WoS database in this paper. We used the advanced search mode. The search keyword search formula was “cancer OR melanoma OR tumour OR tumor OR neoplasm OR cancer” AND “fat OR overweight OR objective OR objective OR compound”. The literature language was English. The search interval was set from 1 January 2003 to 5 November 2022. Figure 3 shows the literature data searched by WoS database and the summary diagram of literature types and subject categories obtained after data summary and data preprocessing. Through downloading summary and data preprocessing, duplicate articles were deleted and 12,111 journals of overseas study were jointly insured. The document types were article (9755, 80.55%), review (1938, 16%), book chapter (67, 0.55%), early access (85, 0.7%) and processing paper (266, 2.2%). The subject categories of literature included oncology (2950), nutrition dietetics (1441), metabolism (1208), public environmental occupational health (980), gastroenterology hepatology (888), biochemistry molecular biology (866), surgery (856), cell biology (730), medicine general internal (598), radiology nuclear medicine medical imaging (562) and so on. As can be seen from the above results, among the research papers on the relationship between cancer and obesity, oncology has made the largest contribution to this research field and produced the most significant amount of scientific research literature. The second is the discipline of nutrition from the perspective of human diet and health, which elaborated the source of cancer. Endocrinology and metabolism have also made great contributions to the research of this field, conducting research on cancer from the perspective of human metabolism and endocrine system. The analysis object of this experiment is the data information of 12,111 academic journals, including academic literature name, author, author institution, national and regional information, keywords, abstracts and references.

### 2.2. Research Methods

#### 2.2.1. Bibliometric Analysis and Knowledge Graph

Bibliometric analysis refers to the cross science of quantitative analysis of all knowledge carriers by means of mathematics and statistics. It is a comprehensive knowledge system that integrates mathematics, statistics and documentation and focuses on quantification. The measurement object of this paper is related to literature in WoS database. Bibliometric analysis can help us quickly understand the development trend and hot topics in related academic fields by means of graph visualization. Knowledge graph is the product of the development of graph data technology. It combines the theories and methods of applied mathematics, graphics, information visualization technology, computer science and other disciplines with the methods of econometric analysis and co-occurrence analysis and displays the core structure, development frontier and overall knowledge structure of the subject by visualization map image, finally achieving the modern theory of multidisciplinary integration. Knowledge graph data visualization integrates information into a single large network that contains a semantic model of data for users to query and explore. Knowledge graph technology converts raw data into graphic information, presenting a more specific and intuitive image. In the knowledge graph, different information is represented by nodes, and the lines between nodes are the associations between information.

#### 2.2.2. Bibliometric Analysis Software

In this paper, CiteSpace (6.1.R3) software was used for bibliometric analysis and data visualization. CiteSpace is a professional bibliometric modeling software, which can carry out systematic, transparent and visual statistics and analysis on existing published research results by quantitative method, with global significance. Furthermore, it improves the subjective preference of researchers, which is inevitable in traditional literature review research. CiteSpace can support many types of bibliometrics research, including institutional co-citation analysis, author cooperative network analysis, keyword hot spot analysis, and topic and field co-occurrence visualization. CiteSpace can show the historical trend of various disciplines and fields and the frontier hotspot of research direction with the help of knowledge mapping technology [35,36]. CiteSpace software can assist researchers in different fields to conduct multivariate, dynamic and complex data information analysis. The knowledge map drawn by CiteSpace consists of a combined network of time-marked co-citation networks, and important knowledge of scientific literature can be identified based on prominent features [37,38]. With the help of CiteSpace, a bibliometric analysis tool, this paper will provide a more valuable review based on the research history of the relationship between cancer and obesity over the past 20 years.

## 3. Results

### 3.1. Number of Annual Journals Published

**Figure 4:**
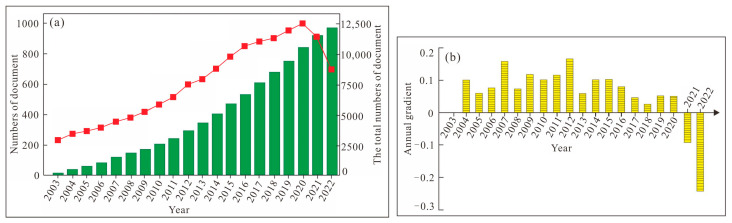
Total amounts and changes of documents on cancer and fat ((**a**). Document publication in 2003–2022. (**b**). ChangeTrend of annual publication of literature.).

With the accumulation of data and the progress in science and technology, literature reviews, as a research method of subject content, are more meaningful than ever before. The number of published documents each year can directly reflect the accumulation of knowledge in a certain research field and the depth of research, as well as reflecting the changes in the amount of scientific knowledge [39,40]. After searching and screening, we retained 12,111 scientific literatures from the Web of Science core collection database from 2003 to 2022. Figure 4 shows the change in the number of articles published each year on the relationship between cancer and obesity and the total number of articles in 20 years. As can be seen from Figure 4, the number of articles published during the period from 2003 to 2022 shows an increasing trend year by year, with a month-on-month change of about 0.1. The figure also shows a small decrease in the number of documents issued in very few years. For example, the number of documents issued in 2021 is reduced compared with 2020, and the number of documents issued in 2022 is also reduced compared with the previous year. The reason may be that the epidemic situation in recent years has caused a relatively big impact on all areas of society, or it may be that the time node for our statistics to publish articles is 5 November, which is still some time away from 31 December 2022. In general, in the past 20 years, the research on the relationship between cancer and obesity in the world has increased year by year, the number of published articles has increased year by year, and the importance of the relationship between cancer and obesity has been rising.

### 3.2. Analysis of Countries and Regions Publishing Papers

**Figure 5:**
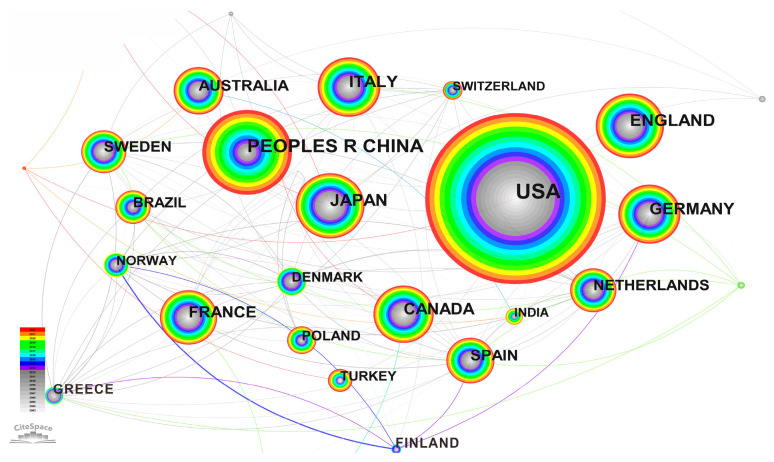
Knowledge graph of published articles by countries and regions.

**Figure 6:**
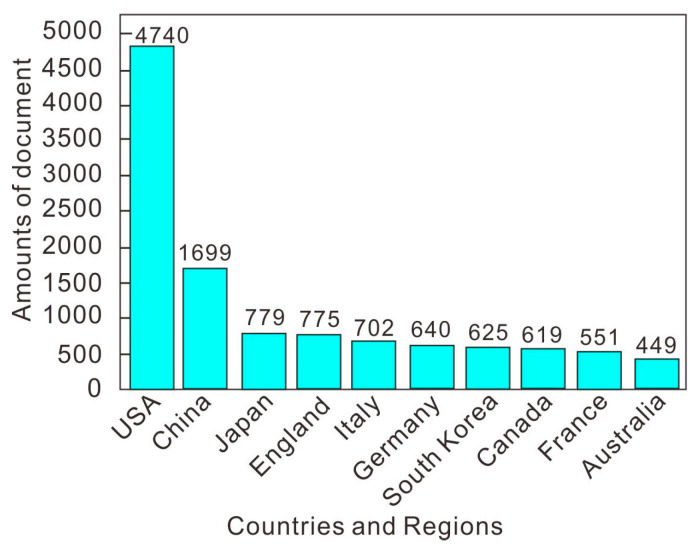
Top 10 countries and regions in the number of published articles.

Experts and scholars all over the world have made outstanding contributions to the research on the relationship between cancer and obesity. The number of articles published by countries and regions in the past 20 years is shown in the Figure 5 knowledge map and the Figure 6 histogram. The United States has the largest number of published articles (4740), accounting for 39.14% of the total number of published articles. The number of articles published in China ranked second (1699), accounting for 14.03% of the total. This shows that the scientific research institutions and scholars in the United States and China have made the largest contribution to the number of published articles in the field of cancer and obesity research. They attach great importance to cancer analysis and prediction and have carried out a large amount of theoretical and applied research, with a view to exploring cancer as a world medical problem. From the connection of the knowledge graph in Figure 5, it can be seen that the articles published in the United States accounted for a large proportion in the 10 years from 2003 to 2012. Most of the articles published in China were concentrated in the 10 years from 2013 to 2022, which indicates that Chinese research institutions have attached great importance to the research on the relationship between cancer and obesity in the past 10 years, and the United States had been studying this field earlier. In the research on the relationship between cancer and obesity, the countries ranked from the 3rd to the 10th are Japan (779), England (775), Italy (702), Germany (640), South Korea (625), Canada (619), France (551) and Australia (449). It can also be seen from Figure 5 that Greece, Spain, Germany, Switzerland and other countries cooperate frequently with other countries in the field of research. Finland and Norway are the two countries with the broadest connection and the closest cooperation. These countries and regions have invested a large amount of funds and budgets in scientific research every year. Among them, China, the United States and Japan are all large countries with large populations, and the annual increase and death of cancer patients are very large, as shown above in Figure 1. In addition, Germany, France, England and Italy are also among the top 10 countries in terms of cancer patients.

### 3.3. Analysis of Authors of Published Articles

**Figure 7:**
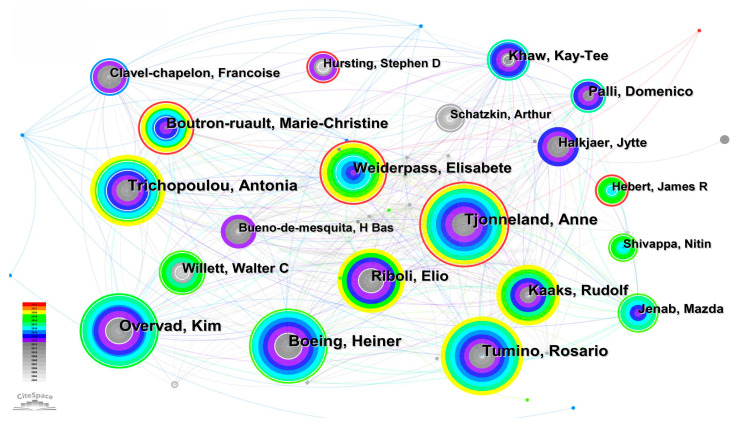
Author knowledge graph.

After the academic papers published by scientific researchers are included in the database, other researchers will retrieve information through the database and quote the article. The more articles published, the higher the frequency of citation and the greater the influence of the author in this field. We use the knowledge map to analyze the number of articles published by authors and the cooperation between authors. Figure 7 is the knowledge graph of authors who published articles. The round node in Figure 7 represents the author. The larger the circle and font, the more documents the author sends. The connection between nodes indicates the communication and cooperation between authors. The thicker the connection, the more documents the author sends through cooperation. Table 1 shows the author information of the top 10 articles published. It can be seen from Table 1 and Figure 7 that Tjonneland and Anne, who published 54 academic papers in the field of the relationship between cancer and obesity in the past 20 years, had the largest number of published articles. Next, is Overvad, Kim, who have published 51 academic papers in this field. Then, Boeing, Heiner (48), Trichopoulou, Antonio (44), Tumino, Rosario (43), Riboli, Elio (40), Kaaks, Rudolf (38), Weiderpass, Elisabet (37), Boutron rault, Marie (34), Khaw, Kay Tee (32). Tjonneland, Anne, Trichopoulou, Antonia, Tumino, Rosario, Weiderpass, Elisabete and others have worked closely with other authors. These authors have carried out a lot of exploration and research in the research on the relationship between cancer and obesity, proving their influence in this field.

### 3.4. Analysis of Published Articles by Institutions

**Figure 8:**
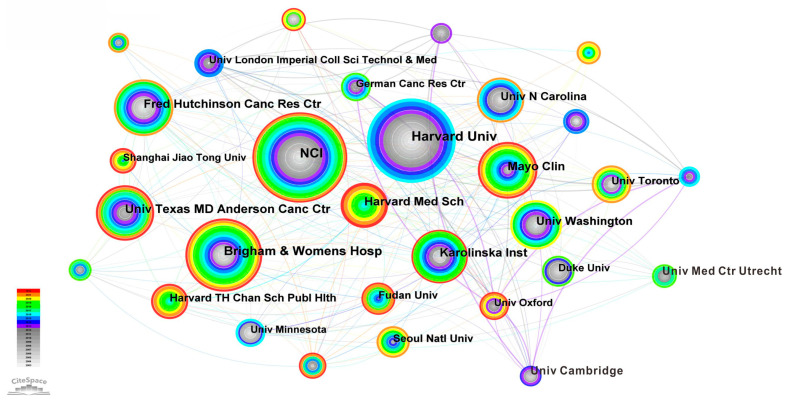
Organization knowledge graph.

We use the knowledge map to analyze the number of articles published by institutions and the close cooperation between them. Figure 8 is the knowledge graph of research institutions. The circular nodes in the figure represent the research institutions. The larger the circle and font, the more articles published by the institutions. The connection between nodes indicates the communication and cooperation between research institutions. The thicker the connection, the more articles published by institutions. Table 2 shows the top 10 research institutions in terms of the number of published articles. It can be seen from Table 2 and Figure 8 that Harvard University in the United States has published the largest number of articles in the research on the relationship between cancer and obesity, and this institution has published 299 academic papers in the past 20 years. Next is the National Cancer Institute, which has published 265 academic papers in this field. Then, Brigham & Womens Hospital (199), University Texas MD Anderson Cancer Center (165), Harvard Medical School (154), Fred Hutchinson Cancer Research Center (145), Mayo Clin (143), Karolinska Institution (139), University of Washington (127) and University North Carolina (109). It can also be seen from Figure 8 that University Cambridge has cooperated closely with other scientific research institutions and published a large number of papers in the past 10 years. The above-mentioned scientific research institutions have made great contributions in tackling the difficult problems in cancer medicine.

### 3.5. Keyword Analysis

**Figure 9:**
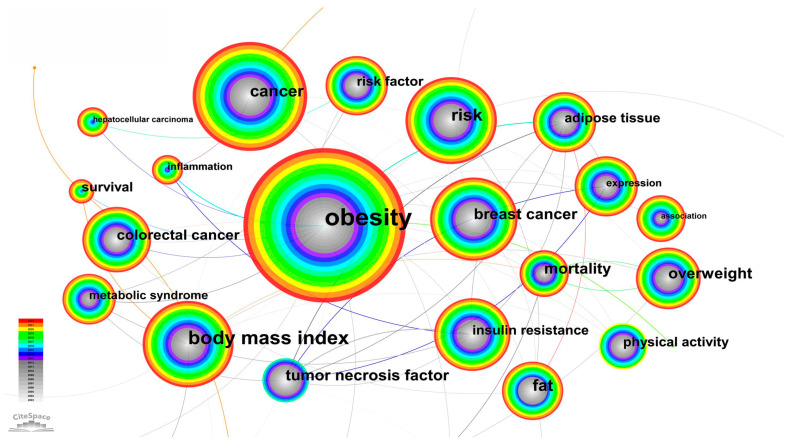
Keywords knowledge graph.

Keyword is a high profile of the research content of an academic journal, which reflects the essence of an article. Through the co-occurrence analysis of keywords in a large amount of the literature on the relationship between cancer and obesity, we can discover research hotspots and development trends in this field [41]. In our research, we set the number of highlighted keywords to 20. Through the keywords analysis function, we obtain the keyword knowledge Graph 9 and the top 20 keywords with the strongest citation bursts Graph 10. We can clearly see in Figure 9 that in the keyword research on the relationship between cancer and obesity from 2003 to 2022, the frequency of occurrence of obesity is the highest, and the associated keywords of cancer, breakthrough cancer, body mass index and risk are also very high. At the same time, popular studies on the relationship between obesity and cancer in the past 20 years also include the women’s breast cancer and body mass index. In addition to the several popular keywords mentioned above, expression, overweight, fat, adipose tissue, risk factor, colorectal cancer, insulin resistance, etc., also appear with high frequency. Tumor necrosis factor and expression, as well as insulin resistance and inflammation, are a group of keywords with high frequency, showing high relevance.

**Figure 10:**
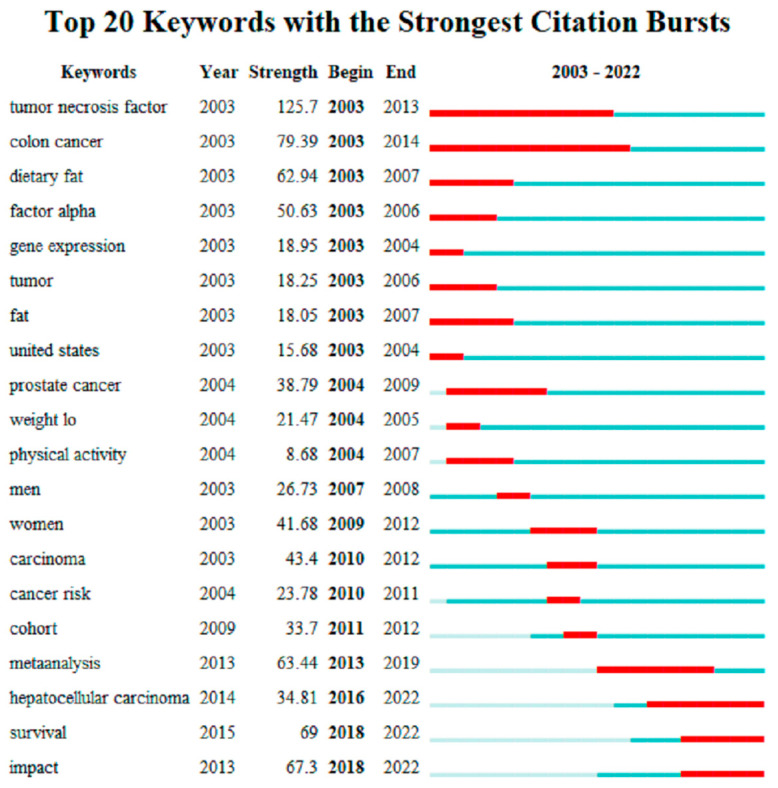
Top 20 keywords with the strongest citation bursts.

As can be seen from Figure 10, the top 20 keywords with the strongest city bursts show that the intensity of the turbine crossing factor is strong, reaching 125.7. Scientific researchers have carried out a lot of research work on the turbine crossing factor in the 11 years from 2003 to 2013. For example, in the literature [42], the authors have performed a lot of experiments and concluded that they showed that tumor necrosis factor alpha led to the hyperthermia-mediated glioma impairment decreasing, which could cause decreases in glioma impairment by promoting the release heat shot factor from neuroblastoma cells.

The authors of [43] established a method to purify anti-tumor necrosis factor from human serum and increase autoantibodies, which can improve the concentration of human anti-tumor immunity to a certain extent. Colon cancer has the longest duration (2003–2014). During this period, researchers have performed a lot of research on colon cancer. For example, the authors of [44] completed a brief review on the medical importance of nutritional drugs and the ability to reduce the risk of colon cancer. Studies have proved that garlic, various antioxidant fruits and vitamins can play a certain role in the anti-proliferation of cancer cells. 

### 3.6. Analysis of Highly Cited Literatures

**Figure 11:**
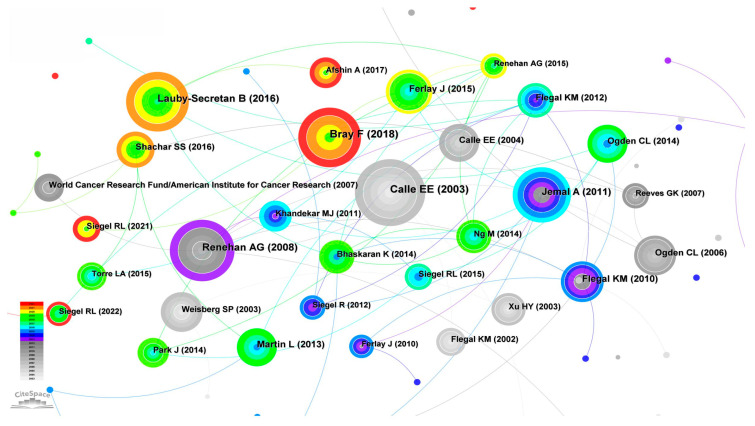
Knowledge graph of highly cited references.

The knowledge base and hotspot of discipline research are the frequently cited references and co-cited networks. Co-cited networks refer to the knowledge networks composed of two articles that are cited by the third or other articles at the same time. When setting the parameters of our knowledge map, we choose the time span from 2003 to 2022, each time period is 1 year long, the node type is selected as cited reference, and the node type is selected as TOP 50 per slice. Figure 11 is the knowledge graph of highly cited references on the relationship between cancer and obesity, which reflects the absorption, utilization and citation of concepts in research frontiers in scientific literature.

It can be seen from the knowledge graph in Figure 11 that Calle EE, Bray F, Lauby secretan B, Renehan AG, Jemal A and others are the top authors cited with the highest frequency, which fully proves their influence in this field. Calle EE published the earliest article, which belongs to the most classic literature in the field. From the analysis, it can be concluded that the article published by the author Bray F. is a relatively new article from 2018, which has been cited 220 times and is also the most classic literature in this field in recent years. The results of the statistical analysis of the first 10 highly cited references are shown in Table 3. In terms of the year of publication, the literature was published and cited from 2003 to 2021, which is the basic literature and classic literature in this field. The articles of Calle EE and Bray F have been cited extensively, with a frequency of 220. Ogden CL has two articles that have been cited in the top 10, including 89 times for articles published in 2014 and 82 times for articles published in 2006. Next, we will briefly introduce the 10 most highly cited articles.

The literature of [45] is the most frequently cited literature. Calle EE, the author of this article, conducted a 16 year large-scale experiment with more than 900,000 people, and calculated the proportion of 900,000 Americans who died of cancer due to being overweight or obese. Finally, it was concluded that weight gain was associated with increased mortality from all cancers and cancers at multiple specific sites. The literature of [46] provides a status report on the global budget of cancer worldwide using the global 2018 estimates of cancer incidence and mobility. The global initiative for cancer registry development can support better estimation and collection of the local data to prioritize and evaluate national cancer control efforts. In the literature [47], the IARC weaving group explained that avoiding weight gain has cancer prevention effects in terms of colon cancer, esophageal cancer, kidney cancer, breast cancer and uterine cancer. Literature [48] conducted a systematic study and analysis on body mass index and incidence rate of cancer and determined that the increase in BMI is related to the increase in the risk of common and uncommon malignant tumors. The literature of [49] believes that a large part of cancer can be prevented and controlled. People can reduce the risk of cancer through tobacco control, vaccination, healthy diet and physical activity. The literature of [50] shows that attractiveness is a risk factor for a variety of chronic conditions, including diamonds, hypertension, hypercholesterol, stroke, heart disease. Certain cancers are also highly associated with obesity. The literature of [51] provided evidence in support of the proposed international consensus definition of cancer cachexia as a multilateral syndrome defined by an ongoing loss of skeleton multiple mass with or without the loss of fat mass. The literature of [52] conducted a systematic analysis of the global cancer incidence rate and mortality in 2012. The most common cancers were lung cancer, breast cancer and colorectal cancer. The most common causes of cancer death were lung cancer, liver cancer and stomach cancer. The article shows that reducing obesity and alcoholism, as well as increasing physical exercise and healthy diet, can reduce the burden of cancer. The literature of [53] and the literature of [54], respectively, analyzed the prevalence of obesity among children and adults in the United States from 2011 to 2012 and from 1999 to 2004. The results showed that obesity prevention remains high in America and the situation is severe.

## 4. Discussion

**Figure 12:**
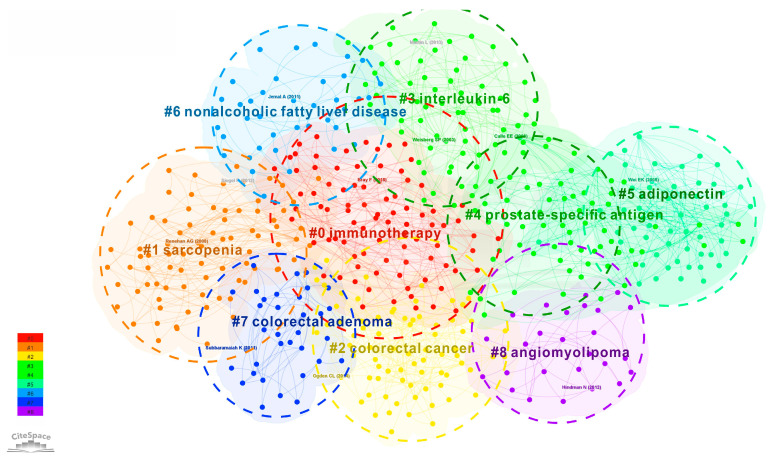
Clustering Knowledge Graph of Research Hotspots (# means cluster).

We have systematically and comprehensively analyzed the scientific research achievements on the relationship between cancer and obesity in the past 20 years through the visualization technology of knowledge graph and found that the relationship between cancer and obesity has always been a topical issue for medical researchers. From macro diet, exercise, body to micro cell molecules and gene expression, these factors related to cancer and obesity have been constantly concerned and deeply studied, gradually forming the knowledge base and topical issues of the discipline. Figure 12 is a map of research hotspots clustering knowledge based on the hot topics of the relationship between cancer and obesity in recent 20 years. We divide the hot spots of the atlas into the following nine clusters for discussion.

Cluster 0 immunity refers to a therapeutic method that artificially enhances or inhibits the immune function of the body to treat diseases in a low or high immune state. Immunotherapy of tumor aims to activate the human immune system and kill cancer cells and tumor tissues by relying on the autoimmune function. In recent years, some scholars have conducted a lot of research on the effect of immunotherapy for obese cancer patients and obtained many innovative theories. For example, in terms of the proposition of the “obesity paradox”, the study in the literature [55] found that cancer patients with elevated subcutaneous fat index (SFI), high body mass index (BMI) and low intramuscular fat index (IFI) had higher survival advantages than ordinary people after receiving immunotherapy. The literature of [56] also has similar research results; the authors’ studies highlighted that elevated BMI was associated with improved outcomes in cases treated by immune checkpoint inhibitor (ICI), which is called the objective paradox.

Cluster 1 sarcopenia is a physiological change in skeletal muscle reduction that gradually appears with age, referring to the decline of human muscle quality and function. The most common causes of sarcopenia are aging, tumor, obesity and malnutrition. Sarcopenia is a common disease in cancer patients. Obese patients should pay special attention to the problem of sarcopenia because the reduction in muscle mass and the increase in body fat will have a very serious impact on the problem of sarcopenia in obese patients. The literature of [57] confirmed through analysis that the proportion of female patients with breast cancer who also suffered from sarcopenia was higher than that of the healthy female population. The use of bioimpedance analysis in association with hand grip strength is relevant for identifying sarcopenia in patients, playing an important role for the patient’s outcome, particularly for early recognition, prompt intervention and periodical reassessment of the risk of sarcopenia and its associated complications. In [58], the authors have proved that age-associated immunity and sarcopenia are integrally connected. Many elderly people with normal body mass index may be obese to varying degrees before suffering from sarcopenia.

Cluster 2 colon cancer is a common malignant tumor of the digestive tract that occurs in the colon. Colon cancer is closely related to people’s diet, and its incidence rate ranks third in gastrointestinal tumors. The common causes of colon cancer are high-fat diet and insufficient intake of human cellulose, which are also closely related to obesity. For example, the literature of [59] evaluated the impact, treatment and prognosis of obesity on colon cancer by reviewing relevant literature on the epidemiology of colon cancer in obese people. The results show that 11% of colon cancer in Europe is caused by being overweight or obese, and obesity may also increase the recurrence or mortality of primary cancer.

Cluster 3 interleukin-6 is an immune cell, which mainly acts on proteins produced by other cells. It can achieve the effect of regulating human immune ability, help to enhance their own immune ability and resistance, and help to improve cancer lesions caused by immune ability. In [60], the authors point out that objectivity is associated with the words breakthrough cancer diagnosis. Through experiments, they proved that objective associated systematic interleukin-6 indirectly enhances preamble atom expression via increased breakthrough cancer cell E2 production. The authors of [61] conducted experimental analysis on liver cancer cells as an example in order to study how obesity increases the risk and development of cancer. The experimental results show that both dietary obesity and genetic obesity are effective initiators of liver tumor cells. The chronic inflammatory reaction caused by obesity and the increase in interleukin-6 may also increase the risk of other cancers.

Cluster 4 prostate-specific antigen (PSA) is a prostate-related antigen and one of the important tumor markers for the clinical diagnosis of prostate cancer. PSA has tissue specificity, which only exists in the cytoplasm of human prostatic acinar and duct epithelial cells, and does not express in other cells. Prostate cancer is the most common cancer in men, accounting for 10–20% of all types of cancer in men. The direct risk factors of prostate cancer include androgen and some metabolic syndrome. The incidence of metabolic syndrome and hormone abnormalities in obese people is very high, so there is a direct correlation between prostate cancer and obesity. The literature in this field includes [62]. The author conducts a correlation analysis of PSA and clinical parameters for obese and diabetes patients, as well as adopting linear regression analysis. The results show that in men with diabetes and obesity, their PSA detection concentration will be significantly reduced, showing a negative correlation. In the literature in [63], the author also obtained that the PSA of obese men is lower than that of normal men of the same age through experiments.

Cluster 5 adiponectin is an endogenous bioactive polypeptide or protein secreted by adipocytes. Adiponectin, as an insulin-sensitizing hormone, can increase and promote the oxidation of fatty acids and the absorption of sugar in skeletal muscle cells and significantly enhance the inhibitory effect of insulin on gluconeogenesis, as well as being an important regulator of body fat metabolism and blood glucose stability. Adiponectin can also inhibit the production and release of the tumor necrosis factor (TNF), which has certain anti-inflammatory effects and an important cytoprotective effect on alcoholic liver injury. In [64], the authors recruited 60 obese people as research objects to evaluate the impact of weight loss on periodontal inflammation. The experimental results showed that the serum adiponectin level of the experimental patients increased significantly after the weight loss plan reached more than 10% and the serum TNF-α. Therefore, controlling obesity is a good way to reduce periodontitis.

Cluster 6 nonalcoholic fatty liver disease (NAFLD) refers to excessive fat deposition in the liver caused by alcohol and other specific factors of liver damage. The etiology is mainly divided into two categories: primary and secondary. Primary is mainly caused by excessive nutrition, such as obesity, hyperlipidemia, diabetes, etc. Secondary diseases are mainly caused by malnutrition, weight loss surgery, drugs or industrial poisoning. The severe condition of nonalcoholic fat liver disease can lead to malignant tumors related to metabolic syndrome, cardiovascular and cerebrovascular diseases and cirrhosis [65]. The study pointed out that the risk of nonalcoholic fatty liver disease is related to the increased risk of liver cancer and most gastrointestinal cancers, especially in patients with early onset. In [66], the authors summarized the relationship between nonalcoholic fat liver disease and obesity, and the pathogenesis of nonalcoholic fat liver disease and nonalcoholic steatohepatitis. The main treatment of NAFLD is to change the lifestyle, including diet, exercise and bad behavior habits.

Cluster 7 colorectal adenoma refers to a prominent lesion on the surface of rectal mucosa protruding into the intestinal cavity, including adenoma, childhood polyp, inflammatory polyp and polyposis. It belongs to a digestive disease. Research [67] shows that, compared with women without diabetes, women with diabetes have an 80% increased risk of colorectal adenoma, and diabetes plus obesity will more than double the risk of colorectal adenoma. Medical analysis suggests that estrogen may affect the growth rate of colorectal cancer, and insulin may also have a direct tumor-promoting effect. In order to study whether visceral fat is a risk factor for colorectal cancer and colorectal adenoma, an experiment on colonoscopy and abdominal fat measurement was conducted on a total of 551 volunteers in the literature [68]. The experimental results showed that the area of visceral adipose tissue was related to the risk of colorectal adenoma.

Cluster 8 angiomyolipoma refers to the pathological changes caused by the abnormal proliferation of adipocytes in blood vessels. It is a vascular tumor with the risk of spontaneous bleeding. Angiomyolipoma usually presents as a benign tumor, which occurs more in the kidney, with a prevalence rate of 0.28% in men and 0.6% in women. The literature of [69] reviewed and studied the angiomyolipoma lesions. The conclusion showed that although the common angiomyolipoma is benign, without proper treatment, it may grow and lead to bleeding, renal dysfunction, venous thrombosis and other complications.

## 5. Conclusions

This paper uses knowledge graph technology to systematically, comprehensively and quantitatively analyze the information in the literature about cancer and obesity in the core database of the Web of Science in the last 20 years. Through the analysis, we learned the source of the knowledge base, research hotspots and research trends in this field, which provided the knowledge base and technical support for medical researchers to continue their in-depth study into the global issue that is cancer. Through this study, we found that obesity can not only affect the occurrence of cancer but also affect the survival and mortality of cancer patients. Obesity-related factors, such as immunity, insulin, adiponectin, adipocytokines and nonalcoholic fatty liver and inflammatory reaction may affect the occurrence of obesity and increase the risk of cancer. Obesity-related cancers include respiratory cancer, colorectal cancer, hepatocellular cancer, prostate cancer, gastric cancer, etc. However, the physiological and biochemical mechanisms of the relationship between cancer and obesity are extremely complex and changeable. Exploring and discovering the molecular and cellular mechanisms between cancer and obesity is still a hot topic at present and will continue to be in the future. Therefore, cancer prevention is still a problem that people should pay attention to in today’s society. Through systematic review and knowledge mapping analysis, the main conclusions of this paper are as follows:

(1) In terms of the number of articles published, the number of articles on the relationship between cancer and obesity published annually around the world shows an increasing trend year by year. The annual change trend is about 0.1, which shows that the research enthusiasm of medical experts and researchers on cancer medical problems is increasing year by year.

(2) As far as the cooperation network is concerned, first, the United States has the largest number of articles (4740). The number of articles published in China ranked second (1699). However, the articles published in China are relatively new, and most of them were published in the decade between 2013–2022. Greece, Spain, Germany, Switzerland and other countries cooperate frequently with other countries in this field. Finland and Norway are the two countries with the broadest connections and the closest cooperation. Secondly, Tjonneland and Anne (54 articles) published the most articles, which proves their influence in the research of this field. The top 10 authors in terms of the number of articles published have contributed a lot of knowledge to the medical research field of the relationship between cancer and obesity, and their cooperation with other authors is also very close. Finally, Harvard University (299 articles) published the largest number of articles in research institutions, which were mainly published between 2003–2015. Harvard Medical School published the latest articles, mostly between 2016–2022. The above countries, authors and institutions have made great contributions to tackling the difficult problems of cancer medicine.

(3) Keywords are used to analyze the research focus on the relationship between cancer and obesity in the past 20 years. From a macro perspective, the main topical keywords include efficiency, body mass index, dictionary fat, physical activity, and nutrition. In terms of micro human metabolism and cell molecules, there are mainly breakthrough cancer, diabetes mellitus, cardiovascular disease, diabetes, etc. From the statistics of key research groups, there are women, obese patients, postmenopausal women, etc. In the past five years, the two key words “survival&impact” have become research hotspots. In addition to focusing on the relationship between cancer and obesity, many researchers and medical experts also pay more attention to the survival rate of patients infected with diseases and the impact of complications.

(4) From the highly cited literature, Calle EE, the author, published an article entitled Excess, objectivity, and mobility from cancer in a prospectively studied corridor of U.S. achievements in 2003, which is a classic literature in this research field and has been cited up to 220 times. In addition, most of the top 10 most-cited literature are reviews, including obesity and cancer mortality, global cancer incidence rate and mortality, body fat, body mass index and cancer, global cancer data statistics, etc.

(5) From the cluster knowledge map of research hotspots, the research direction in this field gradually tends from specific cancer symptoms to the impact of patient survival rate and complications after the disease. Researchers pay more attention to the immune system immunity, immune cells interleukin-6 and adiponectin of human body. In addition, the atlas clustering shows that the research focuses of medical researchers in recent years include sarcopenia, colorectal cancer, prostate specific antigen, nonalcoholic fatty liver disease, colorectal adenoma and angiomyolipoma, which are related to cancer and obesity.

## Figures and Tables

**Table 1:** Top 10 authors.

Number	Count	Year	Authors	Number	Count	Year	Authors
01	54	2009	Tjonneland, Anne	06	40	2007	Riboli, Elio
02	51	2007	Overvad, Kim	07	38	2007	Kaaks, Rudolf
03	48	2007	Boeing, Heiner	08	37	2013	Weiderpass, Elisabete
04	44	2007	Trichopoulou, Antonia	09	34	2009	Boutron-ruault, Marie.
05	43	2007	Tumino, Rosario	10	32	2007	Khaw, Kay-Tee

**Table 2:** Organizations with top 10 articles.

Number	Count	Year	Institutions
01	299	2003	Harvard University
02	265	2003	National Cancer Institute
03	199	2003	Brigham & Womens Hospital
04	165	2008	University Texas MD Anderson Cancer Center
05	154	2016	Harvard Medical School
06	145	2003	Fred Hutchinson Cancer Research Center
07	143	2003	Mayo Clin
08	139	2004	Karolinska Institution
09	127	2003	University of Washington
10	109	2003	University North Carolina

**Table 3:** Classical literature sorted by frequency (the first 10 papers).

Number	Count	Year	Authors	Cited References
01	220	2003	Calle EE [45]	Overweight, obesity, and mortality from cancer in a prospectively studied cohort of U.S. adults
02	220	2018	Bray F [46]	Global cancer statistics 2018: Globocan estimates of incidence and mortality worldwide for 36 cancers in 185 countries
03	186	2016	Lauby-secretan B [47]	Body fatness and cancer- Viewpoint of the IARC working group
04	180	2008	Renehan AG [48]	Body-mass index and incidence of cancer: a systematic review and meta-analysis of prospective observational studies
05	137	2011	Jemal A [49]	Global cancer statistics
06	95	2010	Flegal KM [50]	Prevalence and trends in obesity among US adults, 1999–2008
07	93	2013	Martin L [51]	Cancer cachexia in the age of obesity: Skeletal muscle depletion is a powerful prognostic factor, independent of body mass index
08	91	2015	Ferlay J [52]	Cancer incidence and mortality worldwide: Sources, methods and major patterns in GLOBOCAN 2012
09	89	2014	Ogden CL [53]	Prevalence of childhood and adult obesity in the United States, 2011–2012
10	82	2006	Ogden CL [54]	Prevalence of overweight and obesity in the United States, 1999–2004

## Data Availability

In this study, the authors used the database of Web of Science. https://www.webofscience.com. accessed on 5 November 2022.
